# CT Radiomics and Machine-Learning Models for Predicting Tumor-Stroma Ratio in Patients With Pancreatic Ductal Adenocarcinoma

**DOI:** 10.3389/fonc.2021.707288

**Published:** 2021-11-08

**Authors:** Yinghao Meng, Hao Zhang, Qi Li, Fang Liu, Xu Fang, Jing Li, Jieyu Yu, Xiaochen Feng, Mengmeng Zhu, Na Li, Guodong Jing, Li Wang, Chao Ma, Jianping Lu, Yun Bian, Chengwei Shao

**Affiliations:** ^1^ Department of Radiology, Changhai Hospital, Naval Medical University, Shanghai, China; ^2^ Department of Radiology, No.971 Hospital of Navy, Qingdao, Shandong, China

**Keywords:** pancreatic neoplasm, carcinoma, prognosis, tumor-stroma ratio, multidetector computed tomography, radiomics

## Abstract

**Purpose:**

To develop and validate a machine learning classifier based on multidetector computed tomography (MDCT), for the preoperative prediction of tumor–stroma ratio (TSR) expression in patients with pancreatic ductal adenocarcinoma (PDAC).

**Materials and Methods:**

In this retrospective study, 227 patients with PDAC underwent an MDCT scan and surgical resection. We quantified the TSR by using hematoxylin and eosin staining and extracted 1409 arterial and portal venous phase radiomics features for each patient, respectively. Moreover, we used the least absolute shrinkage and selection operator logistic regression algorithm to reduce the features. The extreme gradient boosting (XGBoost) was developed using a training set consisting of 167 consecutive patients, admitted between December 2016 and December 2017. The model was validated in 60 consecutive patients, admitted between January 2018 and April 2018. We determined the XGBoost classifier performance based on its discriminative ability, calibration, and clinical utility.

**Results:**

We observed low and high TSR in 91 (40.09%) and 136 (59.91%) patients, respectively. A log-rank test revealed significantly longer survival for patients in the TSR-low group than those in the TSR-high group. The prediction model revealed good discrimination in the training (area under the curve [AUC]= 0.93) and moderate discrimination in the validation set (AUC= 0.63). While the sensitivity, specificity, accuracy, positive predictive value, and negative predictive value for the training set were 94.06%, 81.82%, 0.89, 0.89, and 0.90, respectively, those for the validation set were 85.71%, 48.00%, 0.70, 0.70, and 0.71, respectively.

**Conclusions:**

The CT radiomics-based XGBoost classifier provides a potentially valuable noninvasive tool to predict TSR in patients with PDAC and optimize risk stratification.

## Introduction

Pancreatic ductal adenocarcinoma (PDAC) is a challenging disease. Considering all stages of this disease, it has the worst prognosis of all major tumor types in humans, with a five-year survival rate of 9% (1). Surgical resection combined with systemic chemotherapy facilitates the only chance of long-term survival. Moreover, decisions on surgery and adjuvant treatment should be based on an assessment of the tumor stage and surgery-related risks (2, 3). However, patients with similar tumor stages based on the TNM categories have extremely different clinical outcomes (4). This necessitates better biomarkers and tools to predict the treatment response and prognosis, optimize risk stratification, and assist clinicians during decision-making.

The interaction between tumor cells and their microenvironment has gained attention in the past decade. The tumor microenvironment (TME) comprises complex mixtures of non-tumor cells, which play an important role in tumorigenesis, development, metastasis, and drug resistance (5, 6). The tumor stroma promotes tumor progression by producing various nutrients, growth factors, chemokines, and cytokines. Tumor-stroma ratio (TSR) refers to the ratio of tumor cells to the surrounding stroma. Furthermore, it is the most popular macroscopic index that evaluates the TME (7). TSR is reportedly an independent prognostic factor for various solid tumors, including breast cancer (8), lung adenocarcinoma (9), gastric cancer (10), colorectal cancer (11), and pancreatic cancer (PC) (12). Therefore, an evaluation of TSR before decision-making contributes to an accurate risk stratification and facilitates accurate individualized treatment (13).

The evaluation of TSR is usually performed on sections of surgical specimens stained with hematoxylin and eosin (H&E). It is determined by the area with the highest proportion of stroma in the most invasive site (14). Therefore, it is difficult to determine the interstitial state without surgery. Clinicians are unable to accurately evaluate TSR through needle biopsies in patients with advanced PC (15). This can be attributed to the small amount of tissue obtained and the spatial heterogeneity of the tumor. Imaging examination can be an effective and non-invasive method to evaluate the microenvironment of PC. Most related studies have explored the correlation between TSR and conventional imaging parameters in patients with PDAC; however, they did not explore their diagnostic performance (16, 17). Hence, a non-invasive and repeatable method for preoperative TSR evaluation of PDAC is needed.

Medical images can not only reflect the macroscopic characteristics but also the cellular and molecular characteristics of the tissue. In clinical practice, only one- or two-dimensional information that reflects macroscopic characteristics, such as tumor size, location, and attenuation, can be obtained. Radiomics can transform the imaging data into a high-dimensional feature space and use it to describe the tumor phenotype in depth (18, 19). In this study, we used computed tomography (CT) images to extract high-dimensional radiomics features from PDAC, evaluate their relation to PC TSR, and their diagnostic efficacy in patients with PDAC.

## Materials And Methods

### Patients

**Figure 1:**
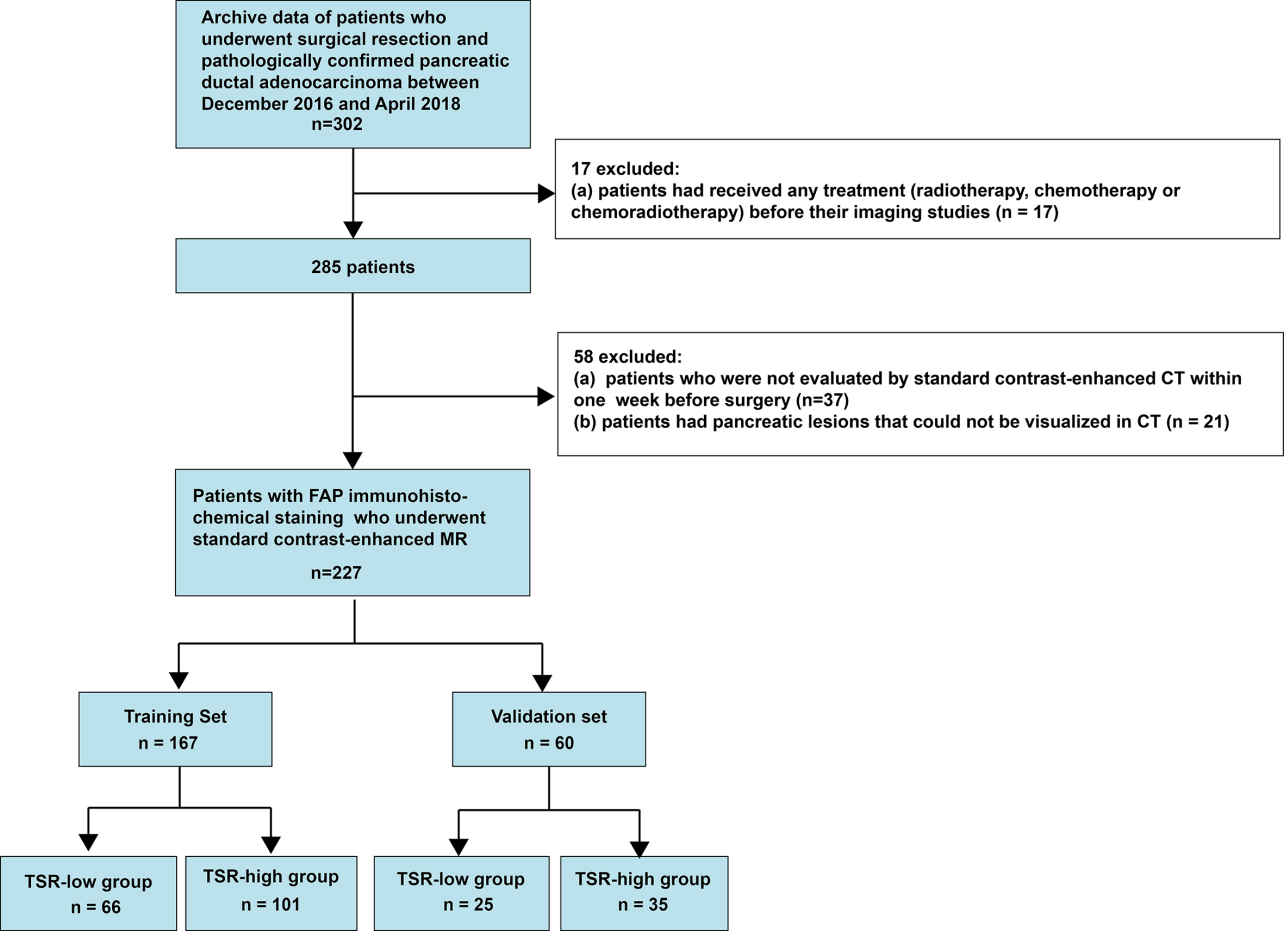
Flow chart illustrating the patient selection process.

This retrospective single-center cross-sectional study was reviewed and approved by the Biomedical Research Ethics Committee of our institution. The requirement of informed consent from patients was waived by the Institutional Review Board. We obtained the data from consecutive patients who had been treated for PC at our institution between December 2016 and April 2018 (**Figure 1**).

The inclusion criteria were as follows (1): patients who had undergone a surgical treatment and (2) patients in whom PDAC had been pathologically confirmed. In contrast, the exclusion criteria were as follows (1): patients who underwent treatment of any type (radiotherapy, chemotherapy, or chemoradiotherapy) before the imaging studies (2), patients who had not been evaluated by contrast-enhanced multidetector computed tomography (MDCT) within a week before the surgery, or (3) patients with pancreatic lesions that could not be observed on MDCT images. Consequently, we included 227 consecutive patients with PDAC, including 151 men (age: 59.14 ± 9.55 years; range: 30-82 years) and 76 women (age: 63.86 ± 7.91 years; range: 40-84 years). The prediction model was developed for a primary set that consisted of 167 consecutive patients admitted between December 2016 and December 2017, including 108 men (age: 58.44 ± 10.00 years; range: 30-82 years) and 59 women (age: 65.29 ± 6.23 years; range: 53-84 years). Thus, 60 consecutive patients, including 43men (age: 60.88 ± 8.15 years; range: 45-79 years) and 17 women (age: 58.88 ± 10.91 years; range: 40-73 years), admitted between January 2018 and April 2018 constituted an independent validation set.

### CT Scanning

We performed multiphasic CT with a pancreas-specific protocol using 320-slice multidetector-row CT scanners (Aquilion ONE, Canon Medical Systems, Tokyo, Japan). The CT parameters were as follows: 120 kV; effective mAs, 150; beam collimation, 160×0.5 mm; matrix, 350×350; and gantry rotation time, 0.5 s. We conducted a non-enhanced CT, followed by a dynamic contrast-enhanced CT scan. The scan delay time was determined according to the test bolus. We injected the contrast agent (90–95 mL of 355 mgI/mL iopromide; Ultravist 370, Bayer Schering Pharma, Berlin, Germany) at a rate of 5.5 mL/s with a power injector (Medrad Mark V plus, Bayer, Leverkusen, Germany) *via* the forearm vein, followed by an injection of 98 mL of normal saline to irrigate the tube. Following the injection, we performed contrast-enhanced CT in the arterial (20-25 s), portal venous (60-70 s), and delayed (110-130 s) phases. The slice thickness/intervals of CT were 0.8/1.0 mm, respectively. The scanning range extended from the level of the diaphragm to that of the pelvis.

### Pathological Image Analysis

We standardized the pathological examination and analysis as described previously (20). We sliced the entire specimen into 5-mm thick sections, resulting in 10–35 (average, 24.5 ± 6.7) formalin-fixed paraffin-embedded (FFPE) blocks for each specimen. Subsequently, we cut each FFPE block into 4-µm-thick sections on whole-tissue glass slides measuring 7.6×5.2 cm^2^. Slides stained with H&E were scanned using a Hamamatsu whole slide scanner (NanoZoomer S60, Hamamatsu Healthcare, Japanese) to obtain digitalized whole-mount slide images (DWMSIs), with an average file size of 6.47 GB. Moreover, we could observe DWMSIs using NanoZoomer Digital Pathology view2 software version 2.7.25. The TSR was determined in all patients with available DWMSIs. We semi-quantitatively assessed the percentages of epithelial and stromal components using the mean value of medium power fields at 100× magnification of the entire tumor scope on DWMSIs (range, 2–3) with a tumor identified at 200× magnification. The TSR was estimated at 5/5, 6/4, 7/3, 8/2, 9/1. Two senior pathologists with 30 and 20 years of experience in pancreatic pathology independently scored the TSR. They resolved any disagreement by discussion. We had determined “5/5 (1)” as the best cut-off value of TSR for prognosis discrimination. Hence, TSR>1 denoted a low stromal component. In contrast, TSR ≤ 1 indicated a high stromal component.

We recorded all pathologic results for the following factors (1): T and N categories, evaluatedon the basis of the American Joint Committee on Cancer TNM Staging Manual, 8th Edition (21) (2); the grade of differentiation (3); duodenal invasion (4); common bile duct invasion (5); lymphovascular space invasion (LVSI); and (6) peripancreatic nerve.

### Radiological Imaging Analysis

We used original cross-sectional arterial and portal venous phase images for the analysis. All images were analyzed by two abdominal radiologists with 30 and 10 years of experience, respectively. They were blinded to the clinical and pathological details. Moreover, the final results were determined by a consensus.

All tumors were evaluated for the following characteristics (1): CT-reported tumor size [i.e., the maximum cross-sectional diameter of the tumor (22)] (2); tumor location: pancreatic head, body and tail (3); pancreatitis identified by the stranding of the peripancreatic fat tissue, ill-defined parenchymal contours, and fluid collections in the peripancreatic region (4); pancreatic duct cut-off and dilation (>3 mm) (5); common bile duct cut-off and dilation (>10 mm) (6); parenchymal atrophy (7); contour abnormality (8); cyst: the presence of pseudocysts and retention cysts; and (9) vascular invasion: an invasion of the common hepatic artery, splenic artery and vein, celiac artery trunk, gastroduodenal artery, superior mesenteric artery and vein, and portal venous vein. The criteria included vessel occlusion, stenosis, or more than half of the perimeter being in contact with the tumor.

### Radiomics Workflow

**Figure 2:**
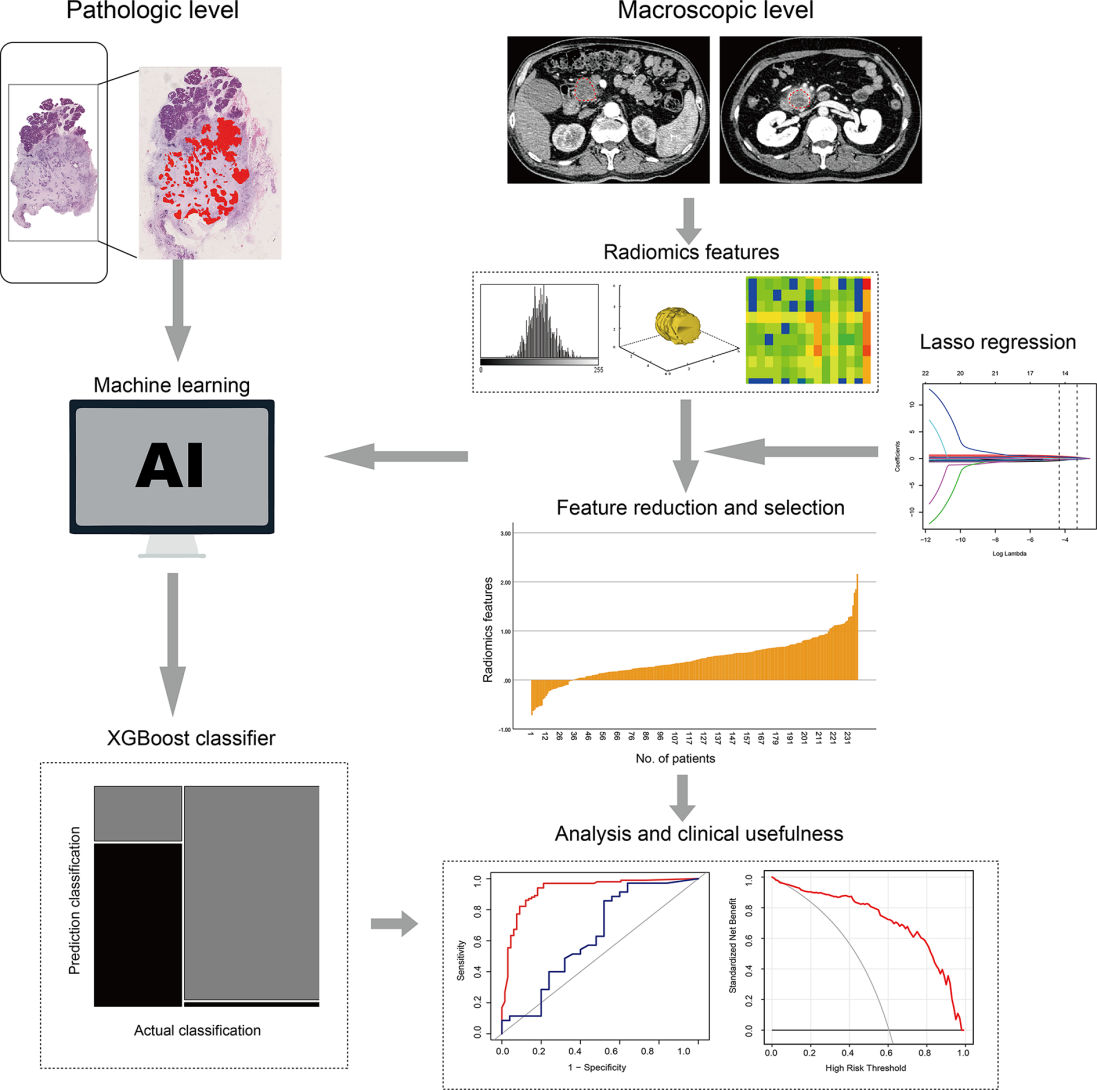
Radiomics workflow.

The radiomics workflow included the following stages (1): image segmentation (2), feature extraction, and (3) feature reduction and selection. The detailed method has been described in a previous study (**Figure 2**) (23).

We used the draw tool, available in the Editor module of 3D Slicer version 4.8.1 (open-source software; https://www.slicer.org/), to delineate the tumors in multiple slices. We extracted the volume of interest for each patient by stacking the corresponding regions of interest (ROIs), delineated slice-by-slice. Radiomics feature extraction was performed using the open-source Python package Pyradiomics 1.2.0 (http://www.radiomics.io/pyradiomics.html) (24). We used the following two classes of feature extraction methods: original feature and filter class. The latter included the following seven categories: logarithm, exponential, gradient, square, square root, lbp-2D, and wavelet. We extracted a total of 1,409 two and three-dimensional features from primary tumors in the arterial and portal venous phase and classified them into seven groups as follows: (a) first-order statistics, (b) shape features, (c) gray-level cooccurrence matrix features, (d) gray-level dependence matrix features, (e) gray-level run-length matrix features, (f) gray-level size-zone matrix features, and (g) neighborhood gray-zone difference matrix features. Feature selection comprised the following three steps: variance analysis, Spearman correlation analysis, and LASSO logistic regression algorithm. Finally, a radiomics score (rad-score) was calculated for each patient *via* a linear combination of selected features that were weighted by their respective coefficients.

Two radiologists (readers 1 and 2) performed the ROI segmentation in a blinded fashion to assess the interobserver reliability. Reader 1 repeated the feature extraction twice during a one-week period to evaluate the intraobserver reliability. Moreover, the reader completed the remaining image segmentations. The readout sessions were conducted over two weeks. The inter- and intraobserver reliability were assessed by obtaining the intraclass correlation coefficient (ICC). ICC values >0.75 were selected for the subsequent investigation.

### Statistical Analyses

We conducted normal distribution and variance homogeneity tests on all continuous variables. While those with a normal distribution are expressed as the mean and standard deviation, those with non-normal distributions are expressed as medians and ranges. We evaluated the overall survival (OS). While deaths were set as events, deaths attributed to other causes were set as censored observations. We calculated survival times from the date of diagnosis to the time of death or the end of follow-up (August 1, 2020). Initially, we classified all patients into two groups, namely TSR-low and TSR-high group. We examined the differences in all variables between the groups. We conducted the student’s t-test (normal distribution), Kruskal-Wallis H test (skewed distribution), and chi-square test (categorical variables) to determine the intergroup statistical differences. The rad-scores were subsequently constructed by the least absolute shrinkage and selection operator (Lasso) regression. Moreover, we constructed the prediction model by extreme gradient boosting (XGBoost). XGBoost was performed using R software supplemented with the XGBoost package. The discrimination of the models was evaluated by the receiver operating characteristic (ROC) curves, and the area under the curve (AUC) was calculated concurrently. We assessed the calibration of the model using the calibration curves and Hosmer-Lemeshow test. Furthermore, we grouped the patients according to the prediction results of the XGBoost classifier. Kaplan-Meier estimates were applied to plot the survival curves, and the log-rank test was performed to analyze the differences between the curves. Moreover, we determined the clinical usefulness of the model with a decision-curve analysis by quantifying the net benefit at different threshold probabilities.

A two-tailed p-value <0.05 was considered statistically significant. All analyses were performed using R software (version 3.3.3, The R Foundation for Statistical Computing, Vienna, Austria).

## Results

### Clinical Characteristics

**Figure 3:**
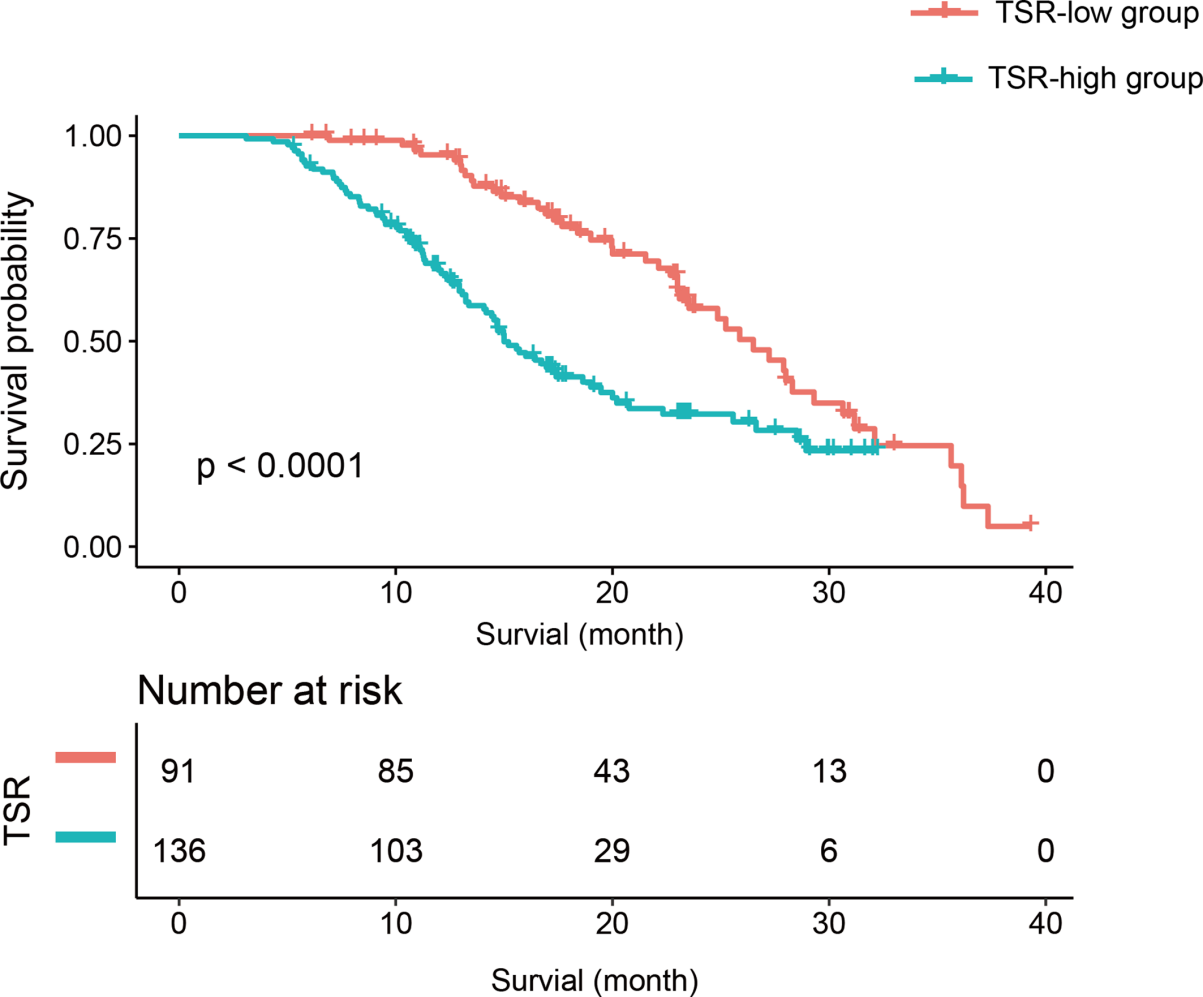
The Kaplan-Meier curve and log-rank test. Patients in the tumor-stroma ratio (TSR)-low group had significantly longer survival than those in the TSR-high group.

There were 91 (40.09%) and 136 patients (59.91%) in the TSR-low and TSR-high groups, respectively. However, 44 and 83 patients had died in the TSR-low and TSR-high groups, respectively. The Kaplan-Meier curves of the two groups were significantly distinct (p=0.002) (**Figure 3**). A log-rank test revealed significantly longer survival duration in the TSR-low group (mean: 25.23 months, 95% confidence interval [CI]: 23.00-35.63) than that in the TSR-high group (mean: 16.43 months, 95% CI: 14.67-20.77). In the univariate analysis, TSR was significantly associated with OS (HR: 2.25, 95%CI: 1.54-3.30, p<0.0001).

Among the clinical, pathological, and imaging characteristics, there were significant between-group differences in the T category in the training and validation set, and bile invasion in the training set. **Table 1** summarizes the patient characteristics.

**Table 1:** Baseline characteristics of patients with pancreatic cancer.

Characteristics	Training set			Validation set		
	TSR-low (n = 66)	TSR-high (n = 101)	*p-* Value	TSR-low (n = 25)	TSR-high (n = 35)	*p-* Value
**Clinical characteristics**						
Sex, n (%)			0.82			0.53
Male	42 (63.64)	66 (65.35)		19 (76.00)	24 (68.57)	
Female	24 (36.36)	35 (34.65)		6 (24.00)	11 (31.43)	
Age, years (meanSD)	60.89 ± 8.91	60.84 ± 9.79	0.97	59.76 ± 9.77	60.71 ± 8.47	0.69
BMI, kg/m^2^ (meanSD)	22.68 ± 2.99	29.31 ± 65.35	0.41	23.58 ± 3.13	22.65 ± 2.73	0.23
Operation, n (%)			0.18			0.36
Pancreaticoduodenectomy	35 (53.03)	64 (63.37)		15 (60.00)	25 (71.43)	
Distal pancreatectomy	31 (46.97)	37 (36.63)		10 (40.00)	10 (28.57)	
**Pathological characteristics**						
T stage, n (%)			0.01			0.03
T1	3 (4.55)	4 (3.96)		1 (4.00)	1 (2.86)	
T2	6 (9.09)	27 (26.73)		4 (16.00)	16 (45.71)	
T3-4	57 (86.36)	70 (69.31)		20 (80.00)	18 (51.43)	
N stage, n (%)			0.35			0.43
N0	35 (53.03)	42 (41.58)		11 (44.00)	12 (34.29)	
N1	25 (37.88)	47 (46.53)		10 (40.00)	20 (57.14)	
N2	6 (9.09)	12 (11.88)		4 (16.00)	3 (8.57)	
Grade of differentiation, n (%)			0.19			0.46
Well-moderately	50 (75.76)	67 (66.34)		18 (72.00)	22 (62.86)	
Poorly-undifferentiated	16 (24.24)	34 (33.66)		7 (28.00)	13 (37.14)	
Duodenum Invasion, n (%)			0.93			0.75
Negative	46 (69.70)	71 (70.30)		16 (64.00)	21 (60.00)	
Positive	20 (30.30)	30 (29.70)		9 (36.00)	14 (40.00)	
Bile Invasion, n (%)			0.03			0.06
Negative	50 (75.76)	69 (68.32)		20 (80.00)	20 (57.14)	
Positive	16 (24.24)	32 (31.68)		5 (20.00)	15 (42.86)	
LVSI n (%)			0.19			1.00
Negative	47 (71.21)	62 (61.39)		15 (60.00)	21 (60.00)	
Positive	19 (28.79)	39 (38.61)		10 (40.00)	14 (40.00)	
Perineural invasion, n (%)			0.53			0.51
Negative	3 (4.55)	7 (6.93)		0 (0.00)	2 (5.71)	
Positive	63 (95.45)	94 (93.07)		25 (100.00)	33 (94.29)	
**Imaging characteristics**						
CT-reported tumor size, cm (meanSD)	3.26 ± 1.36	3.13 ± 1.52	0.56	3.19 ± 1.93	2.92 ± 0.99	0.48
Location, n (%)			0.18			0.36
Head	35 (53.03)	64 (63.37)		15 (60.00)	25 (71.43)	
Body and tail	31 (46.97)	37 (36.63)		10 (40.00)	10 (28.57)	
Pancreatitis, n (%)			0.11			0.39
No	62 (93.94)	87 (86.14)		24 (96.00)	30 (85.71)	
Yes	4 (6.06)	14 (13.86)		1 (4.00)	5 (14.29)	
PD cutoff and dilation, n (%)			0.96			0.84
No	14 (21.21)	21 (20.79)		7 (28.00)	9 (25.71)	
Yes	52 (78.79)	80 (79.21)		18 (72.00)	26 (74.29)	
CBD cutoff and dilation, n (%)			0.43			0.53
No	47 (71.21)	66 (65.35)		17 (68.00)	21 (60.00)	
Yes	19 (28.79)	35 (34.65)		8 (32.00)	14 (40.00)	
Parenchymal atrophy, n (%)			0.18			0.71
No	41 (62.12)	52 (51.49)		16 (64.00)	24 (68.57)	
Yes	25 (37.88)	49 (48.51)		9 (36.00)	11 (31.43)	
Contour abnormality, n (%)			0.98			0.12
No	23 (34.85)	35 (34.65)		12 (48.00)	10 (28.57)	
Yes	43 (65.15)	66 (65.35)		13 (52.00)	25 (71.43)	
Cyst, n (%)			0.34			1.00
No	57 (86.36)	92 (91.09)		22 (88.00)	31 (88.57)	
Yes	9 (13.64)	9 (8.91)		3 (12.00)	4 (11.43)	
Vascular invasion, n (%)			0.21			0.36
No	15 (22.73)	32 (31.68)		13 (52.00)	14 (40.00)	
Yes	51 (77.27)	69 (68.32)		12 (48.00)	21 (60.00)	

BMI, body mass index; LVSI, lymphovascular space invasion; PD , pancreatic duct; CBD, common bile duct.

### Radiomics Analysis

**Figure 4:**
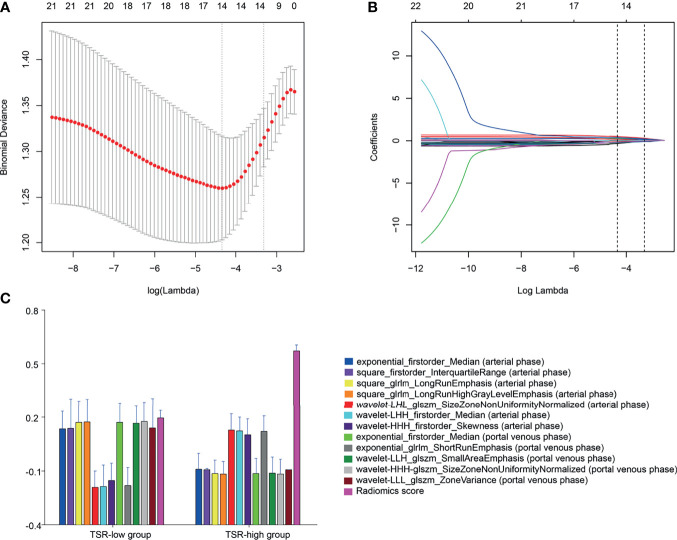
Radiomic feature selection by a parametric method, the least absolute shrinkage and selection operator (LASSO). **(A)** Selection of the tuning parameter (λ) in the LASSO model *via* 10-fold cross-validation based on minimum criteria. Binomial deviances from the LASSO regression cross-validation procedure are plotted as a function of log(λ). The y-axis indicates binomial deviances, whereas the lower x-axis indicates the log(λ). Numbers along the upper x-axis represent the average number of predictors. Red dots indicate the average deviance values for each model with a given λ. The vertical bars through the red dots depict the upper and lower values of the deviances. The vertical black lines define the optimal values of λ, where the model provides its best fit to the data. An optimal λ value of 0.036 with a log(λ) of -3.315 is selected. **(B)** LASSO coefficient profiles of the 25 texture features. The dotted vertical line is plotted at the value selected using 10-fold cross-validation in **(A)** The 12 resulting features with nonzero coefficients are indicated on the plot. **(C)** The error-bar chart of the 12 radiomics features and radiomics score.

In total, 1,409 radiomics features were extracted from portal-phase CT scans. The interobserver ICCs were good, ranging from 0.79 to 0.89. Likewise, the intraobserver ICCs were also good, ranging from 0.80 to 0.91. However, we excluded the radiomics features that did not significantly differ between the groups or did not show significant correlations with TSR expression. The remaining 25 radiomics features were further reduced using a Lasso logistic regression model. We eventually reduced the radiomics characteristics to 12 features (**Figures 4A, B**). Moreover, we used the Lasso logistic regression formula to obtain the rad-score (**Table 2**). The rad-score was significantly lower (p<0.001) in the TSR-high group (median: 0.24; range: -0.72–1.30) than in the TSR-low group (median: 0.52; range: -0.15–2.16) (**Figure 4C**).

**Table 2:** The radiomics features selected by Lasso Regression.

Phase	Prediction model	
Intercept	0.421	
	β	Radiomics name
**Arterial phase**		
	-0.051	exponential_firstorder_Median
	-0.053	square_firstorder_InterquartileRange
	-0.001	square_glrlm_LongRunEmphasis
	-0.078	square_glrlm_LongRunHighGrayLevelEmphasis
	0.252	wavelet-LHL_glszm_SizeZoneNonUniformityNormalized
	0.138	wavelet-LHH_firstorder_Median
	0.086	wavelet-HHH_firstorder_Skewness
**Portal venous phase**		
	0.140	exponential_firstorder_Median
	0.176	exponential_glrlm_ShortRunEmphasis
	-0.133	wavelet-LLH_glszm_SmallAreaEmphasis
	-0.133	wavelet-HHH-glszm_SizeZoneNonUniformityNormalized
	-0.067	wavelet-LLL_glszm_ZoneVariance

Radiomics score =0.421 - 0.051 × exponential_firstorder_Median (arterial phase).

- 0.053 ×square_firstorder_InterquartileRange (arterial phase).

- 0.001 ×square_glrlm_LongRunEmphasis (arterial phase).

- 0.078 ×square_glrlm_LongRunHighGrayLevelEmphasis (arterial phase).

+ 0.252 ×wavelet-LHL_glszm_SizeZoneNonUniformityNormalized (arterial phase).

+ 0.138 ×wavelet-LHH_firstorder_Median (arterial phase).

+ 0.086 ×wavelet-HHH_firstorder_Skewness (arterial phase).

- 0.140 ×exponential_firstorder_Median (portal venous phase).

+ 0.176 ×exponential_glrlm_ShortRunEmphasis (portal venous phase).

- 0.133 ×wavelet-LLH_glszm_SmallAreaEmphasis (portal venous phase).

- 0.133 ×wavelet-HHH-glszm_SizeZoneNonUniformityNormalized (portal venous phase).

- 0.067 ×wavelet-LLL_glszm_ZoneVariance (portal venous phase).

### Apparent Performance of the XGBoost Classifier

**Figure 5:**
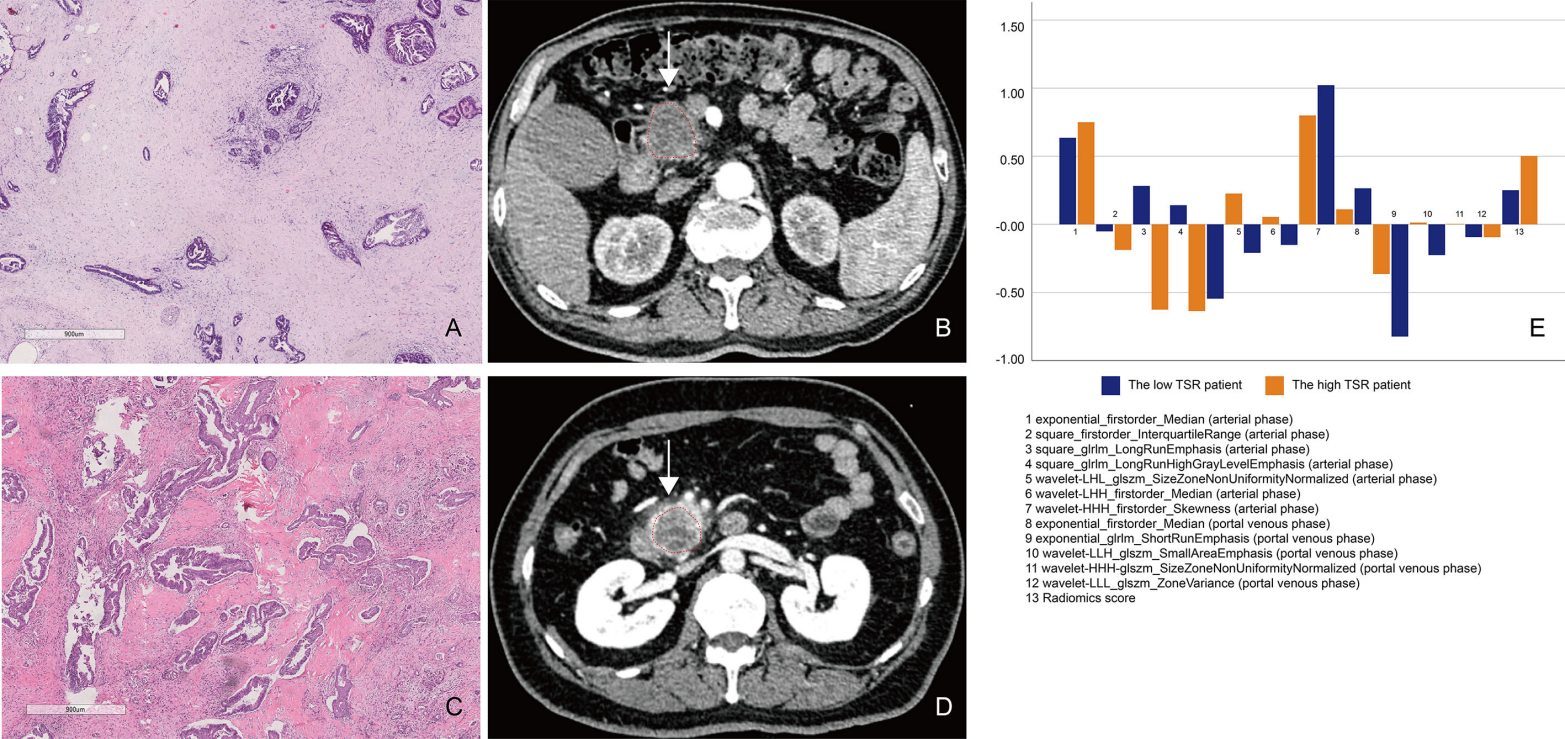
A comparison between patients with low and high tumor–stroma ratio (TSR). **(A, B)** Patient 1: A 69-year-old man with PDAC in the TSR-low group. **(A)** Low TSR expression (×10). **(B)** The axial portal-phase computed tomography (CT) image shows an infiltrative, low-attenuation mass (arrows) located at the pancreatic body and tail. **(C, D)** Patient 2: A case of a 42-year-old woman with PDAC in the TSR-high group. **(C)** High TSR expression (×10). **(D)** The axial portal-phase CT image shows an infiltrative, low-attenuation mass (arrows) located at the pancreatic body and tail. **(E)** The comparison of the 13 radiomics features between patient 1 and patient 2.

**Figure 6:**
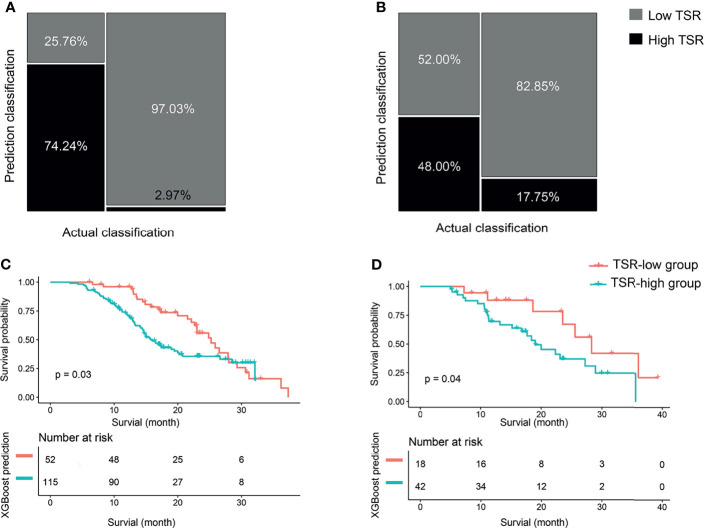
The classification and survival prediction of the extreme gradient boosting (XGBoost) classifier. **(A)** Mosaic plot of the training set. **(B)** Mosaic plot of the validation set. **(C)** The survival prediction of the XGBoost classifier shows significantly longer survival for patients in the tumor–stroma ratio (TSR)-low group than those in the TSR-high group in the training set. **(D)** The survival prediction of the XGBoost classifier reveals significantly longer survival for patients in the tumor–stroma ratio (TSR)-low group than those in the TSR-high group in the validation set.

We developed the XGBoost classifier using the rad-score and tumor size. **Figure 5** depicts the performance of the prediction model. Forty-nine patients were accurately predicted among 66 patients (74.24%, 49/66) in the TSR-low group, whereas 98 patients (97.03%, 98/101) were accurately predicted among 101 patients in the TSR-high group using the XGBoost classifier in the training set (**Figure 6A**). In contrast, 12 patients were accurately predicted among 25 patients (48.00%, 12/25) in the TSR-low group, and 29 patients (82.85%, 29/35) were accurately predicted among 35 patients in the TSR-high group using the XGBoost classifier in the validation set. (**Figure 6B**). The XGBoost classifier predicted an association between TSR and OS (training set: p=0.03, validation set: p=0.04) (**Figures 6C, D**).

**Figure 7:**
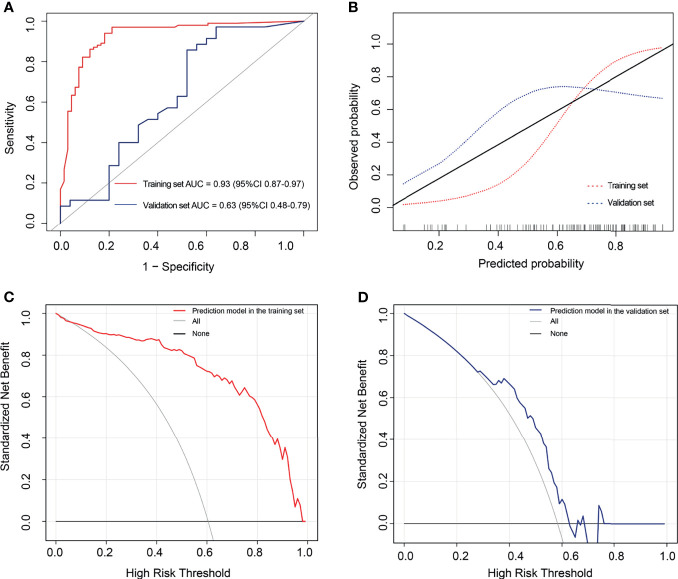
The performance of the extreme gradient boosting (XGBoost) classifier. **(A)** Receiver operating characteristic curves of the XGBoost classifier. **(B)** Calibration curves of the XGBoost classifier. **(C, D)** Decision curve analysis for the XGBoost classifier. The red line represents the training set. The blue line represents the validation set. The gray line represents the hypothesis that all patients had high tumor–stroma ratio (TSR). The black line represents the hypothesis that all patients had low TSR. **(C)** The decision curves in the validation set show that the radiomics score offered greater benefit than the treat-all-patients as low TSR scheme or the treat-none as high TSR scheme in the training set with a threshold probability >0.06. **(D)** The prediction model offered greater benefit than the treat-all-patients as high TSR expression scheme or the treat-none as low TSR expression scheme in the validation set with a threshold probability between 0.29 and 0.63.

The AUC values were 0.93 (95% CI: 0.87-0.97) and 0.63 (95% CI: 0.48-0.79) for the training and validation sets, respectively (**Figure 7A**). While the sensitivity, specificity, accuracy, positive predictive value, and negative predictive value for the training set were 94.06%, 81.82%, 0.89, 0.89, and 0.90, respectively, those for the validation set were 85.71%, 48.00%, 0.70, 0.70, and 0.71, respectively. The curve showed good calibration for the training (p=0.05) and validation sets (p=0.10) (**Figure 7B**).

### Clinical Utility of the XGBoost Classifier

**Figures 7C, D** outline the decision curves of the prediction model. The prediction model offered greater benefit than the treat-all-patients as high TSR expression scheme or the treat-none as low TSR expression scheme, with a threshold probability >0.06 in the training set. Moreover, the prediction model offered greater benefits than the aforementioned expression schemes, with a threshold probability between 0.29 and 0.63 in the validation set.

## Discussion

This is the first study wherein CT radiomics features were used to evaluate the TSR content in the tumors of patients with PDAC. The TSR in this study was more accurate than the traditional method (1). The area of whole-tissue glass slide was 7.6×5.2 cm^2^, which was different from the traditional slide area (7.6 ×2.6 cm^2^) (2). We quantified TSR of the whole slide using DWMSIs, which was different with the microscope at a 100x magnification by the pathologists. The survival duration in the TSR-low group was significantly longer than that in the TSR-high group. Furthermore, we predicted the tumor TSR using the XGBoost classifier that incorporates 12 CT radiomics features and the tumor size. The XGBoost classifier demonstrated favorable discrimination in the training set, but decreased in the validation set.

With the revelation of additional TME mechanisms in determining tumor invasiveness, TSR, as a complete morphological feature of the TME, has been widely confirmed as an independent prognostic factor for various solid cancers. Numerous studies have mentioned that low TSR in gastric cancer, breast cancer, colorectal cancer, and lung cancer, among others, indicate cancer metastasis and poor prognosis (8–11). However, the effect of stromal content on the prognosis of patients with PC is controversial. According to Shi et al. (15) the median OS in patients with stromal ratio >60% was shorter than that in patients with a relatively lower stromal ratio, thus suggesting that stroma is an adverse factor for patients with PC. Joni et al. (25) believed that TSR has no value in evaluating the prognosis of PDAC; however, more studies have reported on high interstitial content (low TSR) being a protective factor for patients with PC. According to the aforementioned studies (12, 17, 26) patients with high tumor interstitial density have longer non-recurrence survival and OS than those with low tumor interstitial density. In this study, the low TSR group had longer survival than the high TSR group. Our results supposedly contradict the tumor-promoting effect of tumor stroma. Therefore, an additional evaluation of the stroma components of the entire tumor can clearly clarify the effect of stroma on the prognosis of patients with PDAC (12). In addition, the TSR-high group was associated with higher T categories, consistent with the study by Li et al. (27). The latter reported on larger tumors with poor stroma than those with rich stroma in patients with breast cancer.

Imaging provides a comprehensive view of the entire tumor and can continuously monitor the development of the disease or its response to treatment (18). Therefore, imaging is a better choice than puncture biopsy for the preoperative evaluation of TSR. Eugene J et al. (28) proposed the delta value (defined as the peak change of the peritumor CT in the parenchymal phase of pancreatic enhancement) in an imaging study of PC. Moreover, they observed that the lower the delta value, the more abundant is the stroma content in the tumor. Shi et al. (15) reported a positive correlation between the high strain ratio (SR) obtained by endoscopic ultrasound elastography and the stromal ratio of PC. Philipp et al. (29) used diffusion-weighted magnetic resonance imaging to evaluate PDAC lesions. The diffusion coefficient was negatively correlated with the percentage of tumor stroma. The aforementioned studies only explored the correlation between TSR and conventional imaging parameters in PC. However, they failed to develop the predicted model. In this study, we developed the XGBoost classifier and determined its discrimination ability and clinical practicability. Moreover, we achieved the results (Training set AUC=0.93, Validation set AUC=0.63). There was a significant decrease in AUC in the validation set, which better reflects the discrimination of the model on novel patients. We think that the following three reasons might have contributed to this. First, the relatively small sample size of the training set may not have contained enough examples to train a generalizable model. Second, overfitting during model training may have resulted in suboptimal generalizability. Finally, the radiomic features we included might not have contained enough generalizable predictors, making the model less capable of generalization. Hence, further large-scale multicenter studies are needed to obtain high-level evidence for the clinical application of the prediction model. In addition, the incorporation of biochemical markers and genetic marker panels into our prediction model could improve its ability to predict TSR in patients with PDAC.

Our research had several limitations. First, we obtained all images using the same CT scanner and imaging scheme in this retrospective study. This, in turn, may limit the generalizability of our study findings. Second, we evaluated TSR in the entire section of tumor specimens, which may overestimate TSR compared to the traditional evaluation method. However, the procedure was supposedly more accurate than the traditional method. Third, to obtain the initial results independent of clinical intervention, we excluded patients who received prior chemoradiotherapy. A previous study showed that preoperative neoadjuvant chemotherapy can affect tumor microenvironment (30). In the future, we will include these patients to further explore the effect of chemotherapy on TSR.

## Conclusion

The CT radiomics-based XGBoost classifier provides a potentially valuable noninvasive tool to predict TSR in patients with PDAC and optimize risk stratification.

## Data Availability Statement

The raw data supporting the conclusions of this article will be made available by the authors, without undue reservation.

## Ethics Statement

The studies involving human participants were reviewed and approved by Changhai hospital. Written informed consent for participation was not required for this study in accordance with the national legislation and the institutional requirements.

## Author Contributions

Conceptualization, CS and JPL. Methodology, YM and HZ. Software, QL. Validation, YB. Formal Analysis, YB. Investigation, FL and XF. Resources, XCF. Data Curation, CM. Writing-Original Draft Preparation, MMZ, YB and YM. Writing-Review & Editing, GDJ, CS and JPL. Visualization, JY. Supervision, NL, LW. Project Administration, JPL. Funding Acquisition, YB, JPL, and CM. All authors contributed to the article and approved the submitted version.

## Funding

This work was supported in part by the National Science Foundation for Scientists of China (81871352), Clinical Research Plan of SHDC (SHDC2020CR4073), 234 Platform Discipline Consolidation Foundation Project (2019YPT001), Shanghai Science and Technology Innovation Action Plan Medical Innovation Research Project (20Y11912500).

## Conflict of Interest

The authors declare that the research was conducted in the absence of any commercial or financial relationships that could be construed as a potential conflict of interest.

## Publisher’s Note

All claims expressed in this article are solely those of the authors and do not necessarily represent those of their affiliated organizations, or those of the publisher, the editors and the reviewers. Any product that may be evaluated in this article, or claim that may be made by its manufacturer, is not guaranteed or endorsed by the publisher.
